# The bridge of iconicity: from a world of experience to the experience of language

**DOI:** 10.1098/rstb.2013.0300

**Published:** 2014-09-19

**Authors:** Pamela Perniss, Gabriella Vigliocco

**Affiliations:** 1 Cognitive, Perceptual & Brain Sciences Department, 26 Bedford Way, London, WC1H 0AP, UK; 2 Deafness, Cognition & Language Research Centre, 49 Gordon Square, London, WC1H 0PD, UK

**Keywords:** language evolution, language development, language processing, iconicity, sign language, co-speech gesture

## Abstract

Iconicity, a resemblance between properties of linguistic form (both in spoken and signed languages) and meaning, has traditionally been considered to be a marginal, irrelevant phenomenon for our understanding of language processing, development and evolution. Rather, the arbitrary and symbolic nature of language has long been taken as a design feature of the human linguistic system. In this paper, we propose an alternative framework in which iconicity in face-to-face communication (spoken and signed) is a powerful vehicle for bridging between language and human sensori-motor experience, and, as such, iconicity provides a key to understanding language evolution, development and processing. In language evolution, iconicity might have played a key role in establishing *displacement* (the ability of language to refer beyond what is immediately present), which is core to what language does; in ontogenesis, iconicity might play a critical role in supporting *referentiality* (learning to map linguistic labels to objects, events, etc., in the world), which is core to vocabulary development. Finally, in language processing, iconicity could provide a mechanism to account for how language comes to be *embodied* (grounded in our sensory and motor systems), which is core to meaningful communication.

## 1. Introduction

This paper provides a new theoretical perspective on three central areas of language study—language evolution, language learning and language processing—based on insights derived from the study of language, spoken or signed, as a system of face-to-face communication. To date, theoretical and methodological approaches to the study of language have been dominated by two main assumptions: (1) that language, as the object of study, is suitably represented in the form of spoken or written words and (2) that the relationship between words and their meaning is arbitrary, determined by convention alone. However, language has developed during phylogenesis as a system for face-to-face communication, it is learnt by infants and children in the context of face-to-face interaction with carers and, for many languages, i.e. spoken languages with no written form and all sign languages, it is always processed in such face-to-face communicative contexts. For both signed and spoken language, recent research has provided evidence that communicative expression comprises the use of different channels in systematic and orchestrated ways (e.g. [1–3] for sign languages and [4–7] for spoken languages), and that language users are sensitive to the semantic and temporal congruence of information expressed in concomitant channels [8–11].

**Figure 1.**
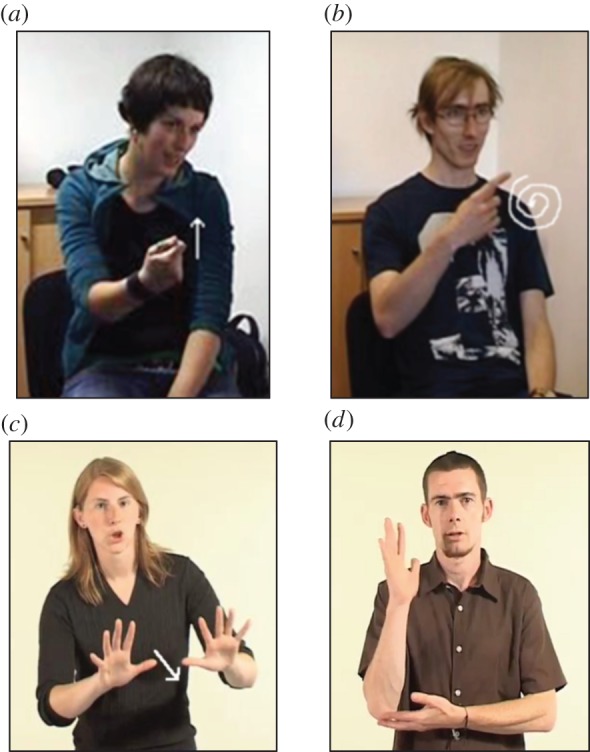
Examples of iconicity in co-speech gesture (gestures accompanying German speech, holding pan (*a*) and entity rotating (*b*)) and in sign language (signs from BSL, PUSH (*c*) and TREE (*d*)).

When we consider language in the context of face-to-face communication, an obvious observation is that language is not simply arbitrary; rather there are multiple iconic (imagistic) cues in communicative/linguistic form to the intended meaning, i.e. properties of communicative/linguistic form often resemble their referent in some way. In spoken languages, speech is accompanied by gestures, as well as facial expression, and the vocal signal may be prosodically modulated. The gestures that accompany speech are often iconic of some aspects of the content of the speech (e.g. figure 1*a*,*b*). Moreover, the prosodic modulation of speech can also provide iconic cues to the meaning (e.g. when a speaker says *looooong* to refer to a long trip, or the sarcasm implied in saying *shoooort*). Finally, iconicity (also referred to as *sound symbolism*) is present in the linguistic signal itself in the form of putatively universal as well as language-specific mappings between given sounds and properties of referents, a propensity that becomes especially visible as soon as we extend our investigation to languages outside the Indo-European family (see [12] for evidence that such sound-symbolic mappings are used by infants and children in vocabulary learning).

In sign languages, perceived visually and produced with the hands, face and body, the potential for iconic forms is much greater—given the modality's affordance for visual-to-visual and action-to-action mapping—and, indeed, across the board, sign languages exhibit a greater degree of iconicity than spoken languages in the linguistic form itself ([13,14]; e.g. figure 1*c*,*d*). Thus, far from being only a very limited phenomenon, iconicity is clearly visible in both signed and spoken languages, on the lexical level and embedded in different channels of expression (e.g. gestural and prosodic expression; see [13] for a review).

In addition to reviewing the evidence for the presence of iconicity across language modalities and typologies, Perniss *et al*. [13] provided a review of the existing evidence that iconicity plays a role in processing and development of both spoken and signed language. Evidence for iconicity effects in these domains continues to accumulate. For example, Thompson *et al*. [15] have recently shown that children learning British Sign Language (BSL) produce and comprehend iconic signs earlier than non-iconic signs. On the basis of such a body of evidence, Perniss *et al*. [13] argue that iconicity is a fundamental property of language, representing an adaptation to a critical constraint on the phylogenesis, ontogenesis and use of language, namely the need to map linguistic form to human (sensory, motor and affective) experience. In this view, iconicity would sit alongside arbitrariness as a fundamental property of language. Specifically, iconicity would be favoured by those processes engaged in ensuring that communication is meaningful, in the sense of related to and grounded in our experience; arbitrariness would, instead, be favoured by those processes engaged in ensuring that the linguistic signal is efficient and discriminable, contributing to exemplar learning and the ability to carry out within-category discrimination [16]. Both the need to map linguistic form to experience and the need for an efficient, discriminable signal are central to successful communication.

In this paper, we spell out the implications of such a hypothesis, which sees iconicity as providing scaffolding for the cognitive system to connect communicative form with experience of the world, for the three core areas of language studies: phylogenesis, ontogenesis and language processing. In phylogenesis, iconicity would help to achieve *displacement*, the ability to refer to things that are spatially and/or temporally remote, and contribute to development of the cognitive ability to maintain conceptual reference. In ontogenesis, iconicity provides a mechanism for establishing *referentiality*, the ability to map linguistic form to meaning, which is at the core of vocabulary learning, as alternative—or in addition—to mechanisms such as correlational (Hebbian) learning and joint attention. In language processing, iconicity is the vehicle for grounding language in neural systems devoted to perception, action and affective experience—in essence, the mechanism by which *embodiment* of language is realized. In arguing that iconicity is a fundamental mechanism that supports language evolution, learning and processing, we provide a unified account of our capacity for language and offer a new theoretical perspective for understanding the cognitive systems and neural substrates underpinning this capacity.

### (a) What is iconicity?

We take iconicity to be any resemblance between certain properties of linguistic/communicative form (this includes sign or spoken language phonology, sign or spoken language prosody and co-speech gestures) and certain sensori-motor and/or affective properties of corresponding referents.

**Figure 2.**
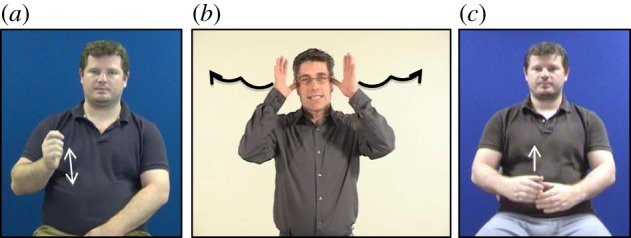
Iconic signs in BSL exhibiting motor iconicity, as in (*a*) the sign HAMMER, depicting the manual manipulation of a hammer; exhibiting perceptual iconicity, as in (*b*) the sign DEER, depicting the shape of a deer's antlers; or exhibiting both motor and perceptual iconicity, as in (*c*) the sign BOTTLE, where the rounded handshape is depictive of the handling of a bottle and the upward tracing movement depicts the cylindrical shape of a bottle.

In sign languages, where all expression is in the visual modality, the potential for iconicity is high and iconic form–meaning mappings are ubiquitous and clearly visible in the lexicon and beyond. Traditional approaches to iconicity in sign languages distinguished between transparent signs (i.e. the meaning is obvious to anyone with shared social/cultural background), translucent signs (i.e. the meaning cannot be guessed by a non-signer, but the motivation for the sign is clear once the meaning is known and a non-signer could choose the correct meaning among alternatives), obscure signs (i.e. the form seems to be iconically motivated, but the motivation has become obscured over time) and opaque signs (i.e. non-iconic signs) [17,18]. Importantly, all iconic signs, even the transparent ones, are conventionalized, a property that sets iconic signs apart from pantomimes and iconic gestures [14,19]. Iconicity can be classified according to whether it is action-based (including iconicity of how to handle an object) or perception-based [20]. For example, many signs are made with handshapes that depict the handling and manual manipulation of an object, as in the sign HAMMER, which is produced as if actually holding and using a hammer (figure 2*a*). Other signs represent salient perceptual features of referents, as in the sign DEER, where the handshape represents the shape of a deer's antlers and the movement of the hands traces the length of the antlers extending from the head (figure 2*b*). In a sign like BOTTLE, the handshape is as if the hand were holding a bottle, but the tracing movement of the hand also provides information about the rounded, cylindrical shape of the bottle (figure 2*c*). Finally, in addition to iconicity in the manual form of signs, iconic mappings in sign language may also be non-manual, through expression on the face and mouth, as in the use of puffed cheeks to indicate roundness or thin, stretched lips to indicate thinness [3].

In spoken languages, the use of the hands in co-speech gestures, and possibly the use of facial expression, offers similar opportunities for iconic representation of action affordances and visual features of referents, and therefore, like signs, gestures can exhibit varying degrees of perceptual/motoric iconicity (e.g. the gesture in figure 1*a* exhibits action-based iconicity, while the gesture in figure 1*b* exhibits perception-based iconicity). Moreover, iconicity exists in the lexicon of all spoken languages in onomatopoeia, evoking acoustic experiences (e.g. *meow, boom, splash*), and, in many languages, extends to other sensory modalities (as in these examples from Japanese: *pika* ‘flash of light’, *tobotobo* ‘a sluggish manner of walking’, *nurunuru* ‘the tactile sensation caused by a slimy substance’, *gorogoro* ‘a heavy object rolling repeatedly’, *korokoro* ‘a light object rolling repeatedly’; [13]). These iconic forms rely on associations between certain sounds and certain qualities of experience (e.g. back vowels corresponding to large or round objects, or to higher intensity of sound or light; front vowels corresponding to small or spiky objects, or to lower intensity of sound or light; voiced consonants corresponding to large objects; voiceless consonants corresponding to small objects). In addition, these spoken language forms rely on correspondences between the structure of the word and features of the event being referred to (e.g. reduplication of syllables corresponding to iterated events). Finally, in vocal prosody, iconicity is achieved by mapping properties of the acoustic signal to properties of an experience, e.g. vowel lengthening to denote an extension or elongation in terms of space (size) or time (duration), as in *looooong* to mean a very long time (see also [21]).

We unify these various manifestations under the single term *iconicity*, regardless of language modality or linguistic tradition. Thus, our use of iconicity subsumes what is typically called *sound symbolism* (as is usually used for spoken languages), including the different terms that refer to word classes exhibiting sound symbolism across different language families (e.g. ideophones, mimetics, expressives and onomatopoeia). Note, however, that our conception of iconicity does not include the notion of non-arbitrary mappings achieved simply through regularity or systematicity of mapping between phonology and meaning (as would be the case, for example, if all words referring to tools differed only in their onset phoneme, cf. [16,22]).

**Figure 3.**
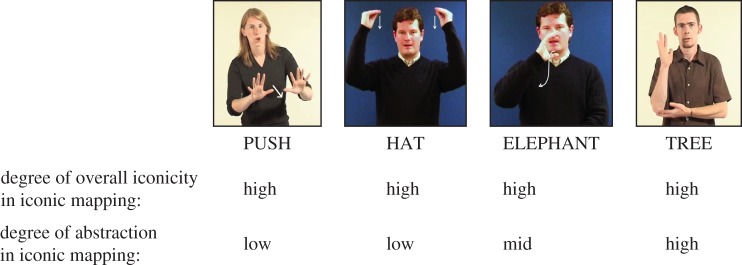
Comparison of ratings of iconic signs in BSL according to overall iconicity of the sign (top) and degree of abstraction or schematization of iconic mapping in the sign (bottom).

Much of current research on iconicity in sign languages has used subjective ratings by native signers on a Likert-type scale as a measure of the degree of iconicity of signs, a method that has proved to successfully predict language acquisition and language processing data [15,23]. However, this holistic notion of iconicity neglects various possible distinctions and, in particular, the fact that the iconic mapping can exhibit varying degrees of *abstraction*. That is, the iconic form can differ in the extent or degree to which it resembles its referent (from more direct to more indirect resemblance). More direct iconic mappings are directly imitative, and thus do not involve a high degree of schematization and abstraction of features of the referent. This is the case for signs like PUSH (figure 1*c*), for example, where the movement of the hands to execute the sign is nearly identical to the movement necessary to perform the actual action of pushing. Similarly, the onomatopoeic word *meow* or an iconic ‘stirring’ gesture accompanying the word *cook* are also directly imitative of their meaning or referent, and these form–meaning mappings thus also do not exhibit high levels of abstraction. Other types of iconic mappings, however, are more indirect and thus more abstract and schematic [14,24]. This is the case, for example, for signs like TREE (figure 1*d*), in which the iconic mapping represents a massive scaling-down in terms of size, and where parts of a prototypical tree are mapped onto parts of the hand and arm. A more indirect, abstract mapping is also exhibited in the examples of Japanese mimetics given further above (i.e. *pika, tobotobo*, etc.). Co-speech gestures may exhibit more abstract and schematic iconic mappings in a similar way. In the vocal modality, words can exhibit varying degrees of abstraction in cross-modal mappings, i.e. where the acoustic signal does not depict an acoustic event. For example, contrast the round mouth in producing *bouba* to refer to rounded shapes/objects to the more abstract mapping of length/gestalt of words corresponding to length/gestalt of events (see [25] for a good review of types of more abstract mappings). It is important to note that ratings of the overall degree of iconicity of signs/words reflect the extent to which any feature of a given sign/word imagistically evokes properties of its referent. Thus, this measure does not coincide with ratings of the degree of abstraction (or schematic complexity) of iconic mappings as described above. This is illustrated in figure 3 for BSL.

The level of abstraction in iconic mappings may be especially critical with respect to the way in which iconicity can be a vehicle for language evolution and development. The more directly imitative iconic mappings may provide the initial point of contact between linguistic form and sensori-motor experience. In scaffolding language development and development of the cognitive system, the facilitatory role of iconicity may depend on starting from the more simple, direct mappings in order to be able to recognize and appreciate the more complex types of iconicity. Interestingly, the fact that the degree of abstraction does not seem to affect performance by adult signers suggests that once learnt, all forms of iconicity support linguistic processing (see also [26]).

## 2. Iconicity, displacement and the phylogenesis of language

The question of language origins is a hot and extensively debated topic engaging researchers from very different fields—biology, psychology, neurology, ethology, anthropology, archaeology and linguistics. Crucial adaptations to language development from a biological perspective include the dropping of the larynx and the direct connection between the primary motor and laryngeal motor cortex [27]. From a socio-cultural perspective, crucial adaptations include tool-making [28] and the development of active sharing, cooperation and teaching among individuals [27,29,30].

Here, our argument is that iconicity also represents a fundamental adaptation. Specifically, iconicity would have played an important part in achieving *displacement*, i.e. the ability to refer to things that are not present in the immediate environment, which is a crucial design feature of language [31]. As we explain below, displacement would have been instrumental in creating the adaptive niche that propelled early hominins from systems of communication based on functional reference and symptomatic signalling to a system based on conceptual reference and deliberate, intentional message transmission [32,33]. The argument rests on the idea that the social structure and cultural development that existed in early hominin groups gave rise to the need to refer to things that are spatially and temporally removed, and that this need—and iconic signalling as one response to it—is a harbinger of conceptual reference. Thus, iconicity would have been instrumental in bringing about the transition from the use of purely functionally referential signals to the use of conceptually referential signals [33–35]. (Some people have argued that this is better achieved in the manual modality because it lends itself better to the production of iconic, motivated signs [36–38]; see also [39].) Below we first discuss the distinction between functional and conceptual reference and the conditions that might have played a key role for displacement to emerge. We then introduce how iconicity might have played a significant role in the development of displacement in communication.

### (a) Functional versus conceptual reference

Many animal calls, e.g. the calls produced by vervet monkeys [40] or even by male domestic chickens [41], are functionally referential in that their function is to pick out a certain class of predator. In the case of vervet monkeys, calls distinguish between different kinds of predators (those in the sky, undergrowth or ground). They are uttered upon perceptual recognition of a predator type and alert other group members to engage in the appropriate flight response. While these and other animal calls provide evidence of categorization of different predator types, the calls can be produced only symptomatically, as a direct reaction to a perceived threat. Thus, there is no evidence here for any kind of conceptual reference—the predator is not actually being labelled based on a mental representation of the referent (cf. [33]). By contrast, when we use words to refer to things, we do so through actual naming, based on a conceptual representation of the things referred to. We can retrieve information about objects and events independent of their immediate presence and our physical perception and experience of them, and are thus not bound to utterances that are purely indexical and symptomatic. This is the crucial difference between functional reference and conceptual reference. Conceptual representation is itself a form of displacement: the representations we have in our minds exist independently of—and thus displaced from—the objects and events they refer to.

### (b) Biological and socio-cultural preconditions for displacement

What are important conditions, in terms of biological development and social and cultural complexity, that would have had to be in place for the need to refer to the not here-and-now to have arisen—and thus for iconicity to have played a role in achieving the ability for displaced reference? One very important condition seems to be group size. Dunbar [42] has argued that brain size is correlated positively to group size, such that even Neanderthals would likely have lived in groups of over 100 individuals. Social group size is intimately linked to cultural development and to the development of complex social structures, where individuals maintain a multitude of social relationships. One major consequence of socio-cultural advancement would be the development of a division of labour among individuals. An important benefit of a division of labour is an enhanced ability to transmit cultural skills (e.g. tool-making skills). As Dediu & Levinson [43] (p. 9) note, citing Henrich [44], ‘One possible reason for the cultural limitations of small populations has to do with the transmission fidelity of culture, with only larger populations having the variance and division of labor to maintain the quality of skills’.

Another important consequence of complex social structure would be the emergence of cooperative information sharing. Factors like mutual inter-individual reliance, management of different social relationships and division of labour would help provide the impetus for cooperative information sharing. Cooperative interaction, and engagement in joint-attentional, information-sharing situations, are distinctively human behaviours [30]. Related to this is an important development in the morphology of the human eye. Humans are the only primates with white sclera and irises small enough for the position of the pupil/iris against the sclera to be clearly visible. This distinctive feature has led to the *cooperative eye hypothesis*, which holds that the human white sclera evolved to make gaze following possible while engaged in joint activities or shared attentional situations [45,46]. The ability to follow the direction of eye-gaze, instead of the direction in which the whole head is turned, is specific to humans, and the specific morphology of the human eye is argued to have evolved to support cooperative social interaction [46].

### (c) How iconicity contributes to displacement

Above, we have presented arguments for cultural developments like division of labour, mutual dependence and cooperative information-sharing emerging in the wake of large groups and complex social structures. One can imagine that the existence of mutual reliance for food and labour across the members of a group engenders the need to refer to things in the not here-and-now. As Kendon [33] (p. 213) puts it, if the division of labour within a group ‘were to involve a periodic *spatial* separation of group members who are otherwise dependent on each other, [c]ommunication about matters not jointly present may thus become necessary’. In Bickerton's [32] scenario, for example, such communication would be necessary for megafauna scavenging, specifically for the recruitment of group members to the remote (i.e. displaced) site at which the animal (carcass) had been discovered.

The use of iconicity, i.e. of imagistic, imitative representations of real objects and actions with objects, would be a key component in achieving displaced reference. For example, in attempting to communicate to someone else the intention to go hunting, one could rely on conceptual traces of previous sensori-motor experiences in hunting, using the face, hands, body and vocal chords to imitate what can be retrieved of these previous sensori-motor experiences to convey the intention to hunt. In this scenario, iconicity is imitative of something that is not there, to evoke some ‘trace’ of a previous experience and to thereby make the event present in a sense. In this lies a seed of conceptual reference, with iconicity bridging between a referent in the world and a representation in the mind, and thereby achieving displaced reference. It is plausible that it is especially the more direct, imitative type of iconicity that would have played a greater role at the beginning, while more complex mappings (e.g. in which the hands give a schematic of an object, as in the BSL sign TREE; figure 1*d*) would have appeared later, with continued conceptual development and therefore development of the ability to abstract from sensori-motor experience. In addition to increased complexity, repeated and frequent use of (iconic) mappings within a community—with feedback to enable grounding and memorization of representations [47]—enables signal reduction and ritualization, leading to form conventionalization and, ultimately, to higher levels of abstraction [47–49]. While we wish to accord iconicity an important, instrumental role in the evolution of language, we do not mean to suggest that iconicity would have been the only factor contributing to the development of conceptual reference. Growing complexity within the socio-cultural structure of hominin groups, for example, with individuals engaged in tool-making and other technical skills and maintaining a multitude of social relationships, would also contribute to the development of more abstract, conceptual thinking. Even assuming that conceptual reference developed under the influence of multiple forces, iconicity would nonetheless be key to language evolution, as we have argued above.

It is clear, in any case, that this scenario relies on the development of storage and retrieval capacity of previous experience in the brain. Importantly, as brain size increased in protohominids, so did brain connectivity. For example, compared to other mammals, primate brains are packed with an extraordinary amount of neurons in relation to the size of their brains. Cortical neurons in primate brains are comparatively small, which means that cortical cells can be densely packed and allow fast communication [50,51]. In addition, this scenario relies on the capacity to recognize another's action (as potentially one's own). The mirror neuron system, by which individuals recognize actions by others because the same neural activation necessary to produce an action is generated by observation of the action, is crucial in this regard [52,53]. For Rizzolatti & Arbib [53], the core of language lies in the development of a proto-dialogue between two individuals based on mutual action recognition through concerted activation of the mirror neuron system. In this account, however, there is no basis for why an individual would come to recognize another's action as an *intentional* communicative signal. Bringing iconicity and the need for displacement, as a result of socio-cultural advancements, into the picture provides an explanatory basis for the communicative intentionality of signals as it removes the necessary, purely symptomatic coupling between a signal and an event, allowing instead the representation (and hence communication) of a concept held independently in the mind.

## 3. Iconicity, referentiality and the ontogenesis of language

It is generally agreed that infants learn their first words through the co-occurrence of a heard word (or seen sign) and a visual scene. Standard approaches assume that the central problem is to explain how children manage to learn labels that are linked only arbitrarily to referents and how they are able to make correct form–meaning associations despite the ambiguity of everyday visual scenes that contain multiple referents [54–58]. Standard solutions to this twofold problem of referential ambiguity—i.e. arbitrary mapping and multiple possible targets—assume a host of *a priori* cognitive skills that the infant brings to the task of word learning, including expectations that words highlight commonalities between objects in the world and that different types of words refer to different types of commonalities [58,59], the capacity to make inferences about the communicative intentions of speakers [60–62] and the ability for statistically driven cross-situational learning [54,56,63].

Recent alternative approaches advocate a closer coupling between perceiving a word (or seeing a sign) and perceptuo-motoric access to a specific referent [64–67]. For example, Yu & Smith [66] argue that toddlers reduce referential ambiguity through their own actions, by coordinating their body, hands and eyes to visually isolate, and specifically zoom into, a given object. Initial word learning would be most effective when labelling by carers occurs during these moments of referent-specific visual attention—and it would seem that carers outside of the laboratory would be especially given to producing labels during such moments. Glenberg & Gallese [67] propose that joint attention guides the process of learning to associate the sensori-motor linguistic processes of hearing and saying a word (and presumably seeing and producing a sign) and the sensori-motor experiences of seeing and holding/using an object. Finally, research suggests that pointing gestures (both by the child and the carer) may also provide a powerful tool for reducing referential ambiguity [68,69].

What remains common to all these approaches is the assumption that labels are only arbitrarily linked to referents in the sensori-motor experience of infants. Hence even when a single referent has been successfully visually isolated, establishing referentiality implies temporal overlap between attention to the (single) referent and exposure to the verbal label (spoken or signed), so that linguistic form and meaning can be linked via Hebbian learning, or other related mechanisms [67,70].

Here, we propose that iconicity provides an additional, critical mechanism for reducing referential ambiguity and therefore for promoting word/sign learning. Moreover, because iconicity provides a learning mechanism that does not require a referent to be present in the immediate visual environment, it also allows for language learning episodes when the objects are *not* present. On this account, the child makes use of a resemblance relationship between form and referent to link linguistic and conceptual form. The presence of iconicity in the input to a child would thus help the child to bridge the gap between experience of the world and the ability to communicate about this experience. As such, similar to the infant's own actions in visually isolating referents, iconicity provided by carers in the input would offer another type of ‘external sensory-motor solution’ ([66], p. 244) to the task of word learning. Of course, for this hypothesis to be viable, there must be evidence that infants and children are sensitive to iconicity and that iconicity is indeed found in the input from carers. Below we review the available evidence.

### (a) Infants' and children's sensitivity to iconicity

For spoken language, several studies have provided evidence that infants (four-months [71,72]) and toddlers (2–3 years [73,74]) are sensitive to sound–meaning correspondences, particularly sound–shape correspondences of the *kiki-bouba* type. (Imai & Kita [12] provide a comprehensive review of the literature concerning the role of sound-symbolic mappings in learning a spoken language.) These findings have been interpreted as suggesting that aspects of iconic, sound-symbolic mappings are universally and biologically grounded.

However, in general, it is argued that effects of iconic mappings do not emerge until about 3 years of age when children develop cognitive awareness of iconicity as a tool to link form to meaning [20,75,76]. This seems corroborated by the finding that children do not start producing iconic gestures until the age of about 2.5 years [77]. The contradiction implied by these two lines of evidence may be resolved by considering the degree of abstraction required by different types of iconic mapping.

**Figure 4.**
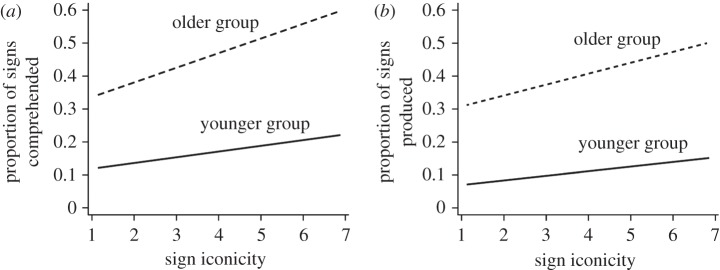
Proportion of BSL signs (*a*) comprehended and (*b*) produced by children in younger (11–20 months) and older (21–30 months) age groups as a function of sign iconicity, as rated on a scale from 1 = *not at all iconic* to 7 = *highly iconic*. (Reprinted from [15] with permission.)

For the acquisition of sign languages, iconicity has historically been treated as unimportant. The initial need to establish recognition of sign languages as fully-fledged natural human languages meant moving the focus away from features of signed language that suggested a pantomimic nature, and proving the existence of linguistic structures and categories equivalent to those in spoken languages in all respects [17,78–80]. This also meant that theoretical assumptions about the fundamental arbitrary nature of language remained intact. However, Thompson *et al*. [15] provided first evidence for a role of iconicity in vocabulary learning in BSL. They showed that the iconicity of signs (operationalized as subjective ratings by adult native signers, see [81]) predicted sign production and comprehension by deaf infants and toddlers (aged 11–30 months), as reported in the BSL Communicative Development Inventory (BSL-CDI) [82]. Interestingly, these authors further reported that the advantage for iconic signs increases with age such that although both younger (11–20 months) and older (21–30 months) children produced and comprehended more iconic than less iconic signs, older children showed a greater effect of iconicity (figure 4). One possible explanation for the difference between younger and older children might be linked to the level of abstraction in the iconic mappings of the signs. The younger children may not have been able to process more abstract forms of iconicity that were available to older children (and who thus showed an effect of iconicity for a greater number of signs.)

In line with this argument, Tolar *et al*. [20] reported that hearing children aged 2.5–5 years learning signs from American Sign Language (ASL) were sensitive to iconic cues from age 3, although at 2.5 they already showed effects of action-based (but not perception-based) iconicity. This suggests that the difference between action-based and perception-based iconicity may be particularly relevant in terms of the developmental time course of access to different types of iconic mapping. In particular, action-based iconicity may be available earlier because it is based more on imitative resemblance (as in PUSH, figure 1*c*), while perception-based iconicity may be available later as it requires more abstract mapping of features (as for example in DEER, figure 2*b*, where the head of the signer needs to be mapped to the head of the animal and the signer's hands need to be mapped to the deer's antlers). However, to our knowledge no existing study has directly addressed action-based versus perception-based iconicity, or—possibly more importantly—the level of abstraction in the iconic mapping in the acquisition of a sign language as a first language.

### (b) Iconicity in the input to infants and children

To date, we know little about how iconicity is conveyed in carers' input to children. Are iconic mappings conveyed systematically in multiple channels of expression? Do carers explicitly use different channels to highlight resemblance relationships between communicative form and referents in the world, i.e. referents in specific joint-attentional situations?

More is known about non-iconic modifications and multichannel combinations of the language input. For example, for spoken language, there has been a considerable amount of research on the ways in which carers modify their speech when interacting with infants and toddlers—typically referred to as ‘motherese’ or ‘infant-/child-directed speech’ [83]. These modifications have been found to exist cross-linguistically and cross-culturally, and include higher pitch, shorter utterances, longer pauses, and exhibit generally exaggerated and more repetitive intonation [83,84]. Functionally, they have been described as engaging attention, maintaining arousal, and facilitating segmentation and processing of the signal. Similar modifications have been found in the motherese of signed language [85–88]. For example, Masataka [87] found that deaf mothers using Japanese Sign Language exhibited more exaggerated movements, more repetition and bigger, slower signing when interacting with their deaf infants (aged between 8–11 months) compared to when signing with deaf adult friends.

**Figure 5.**
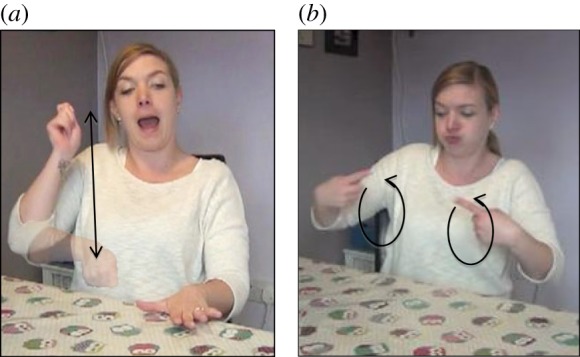
Examples of iconic modification in BSL, showing manual modification in (*a*), where the action affordance of a hammer is exaggerated in the sign HAMMER, and showing modification on the face/mouth in (*b*), where the vibrating lips reflect the spinning motion of tires.

There is some initial evidence that carers do modify their language in terms of the amount and type of iconicity conveyed when speaking with children versus adults or when conveying information about referents that are absent versus present in the communicative context. For spoken language, Saji & Imai [89] found that Japanese-speaking carers used more sound-symbolic and onomatopoeic words when speaking to their toddlers than when speaking to adults (see also [12]). In sign languages, where iconicity is ubiquitous in the lexicon, features of referents reflected in the iconic mappings of signs may be similarly exaggerated in child-directed signing. Perniss *et al*. [90] found that deaf adults, asked to imagine playing with their children, embedded more iconicity into their signing when toys were absent compared to when toys were present (figure 5). The comparison between conditions in which referents are present versus absent is important given that parents do talk about things that are not in the here-and-now with their children and, as argued above, it is in these contexts that iconicity can be especially useful in reducing referential ambiguity. As such, iconicity may provide a broadly applicable and flexible learning mechanism.

However, an extensive literature looking at speech + gesture combinations in spoken language suggests a different picture, indicating that iconic gestures may not play much of a role in language development [77,91–96]. These studies (looking at children in an age range between 14 and 42 months) have found that vocabulary size is predicted both by children's own use of gesture as well as by the amount of gesture in the parental input [95], but have found that over time, the frequency and distribution of functionally different types of speech + gesture combinations (i.e. disambiguating, re-inforcing or supplementary) remains stable in carers' input, and changes only in children, presumably reflecting changes in cognitive skills. These studies mainly emphasize the role of gesture production by children in eliciting labelling from their parents [92] and in predicting language development in the children [77,93,95]. Crucially, these findings suggest that children's communicative milestones in integrating speech and gesture are not the direct result of the nature of gestural input received [94], and generally indicate a preponderance of deictic (pointing or showing) gestures compared to only a small proportion of iconic (or representational) gestures [94,97].

This latter fact, however, may be the result of scoring decisions by the researchers. Iconicity may be embedded in certain kinds of deictic gestures, but may go unreported. For example, the category of deictic gestures used by Puccini *et al*. [98] includes *Action Demonstration* (with an object)*, Object Demonstration* (with an object) and *Show*. These types of deictic gestures seem very amenable to the embedding of iconic elements. For example, a parent could have been observed performing an action or object demonstration consisting in holding a toy frog and moving the frog in an iconic, jumping manner through the air while providing the label ‘jump’ or ‘frog’. However, this would have been coded as deictic, not as iconic. Thus, in focusing on a (broad) category of deictics, and possibly subsuming iconic elements under this category, the role of iconicity in language learning may be obscured and perhaps unfairly dismissed [99,100]. In another study, Gogate *et al*. [91] compared speech and gesture combinations in teaching novel nouns and verbs to infants, focusing on pointing and showing gestures. They found that carers included more movements with ‘show’ gestures when teaching novel verbs to infants compared to novel nouns. This invites the speculation that the movements involved in these ‘show’ gestures were related in some way to manner of movement of the referents, and that carers may have created an iconic mapping to promote word learning in their infants. (The use of iconicity to convey verb-like meanings furthermore suggests that the role of iconicity in language learning may extend beyond the object level to verb and event-level learning, providing an alternative/additional mechanism to syntactic bootstrapping in verb learning [101].)

However, differences in the role of iconicity in the manual components of signs and in co-speech gestures may also be related to whether iconicity is expressed in the primary or secondary linguistic channel. Hands provide primary information in sign languages, but only secondary information in spoken languages where, instead, speech is the primary source of information. It is the case that for spoken languages, the (limited) evidence suggests a role of iconicity in speech through the use of sound-symbolic mappings [12,73,89].

## 4. Iconicity, embodiment and language processing

In the past two decades, a growing body of literature has provided support for the idea that understanding language involves engaging in *simulations* of corresponding sensori-motor experience (e.g. [102–104]). The current evidence suggests that it is unlikely that language processing engages in full the same systems that are engaged in actual sensori-motor experience with the physical world (as a strong embodiment view would predict [105,106]), but, in general, the evidence is compatible with views that higher level sensori-motor processes are engaged whenever we process language referring to sensori-motor experience (see [107] for a comprehensive review of the neuroscientific evidence). With few exceptions [67], studies have not addressed how this may come to be, or in other words, few studies have endeavoured to identify the explicit mechanisms that underscore the coupling between language processing and sensori-motor processing. One reason for the lack of such studies may well be that, assuming arbitrary links between linguistic form and meaning, researchers more or less implicitly assume that such coupling must be realized during language development as a Hebbian type of association (see also [70,108]). As Glenberg & Gallese [67] propose, in language acquisition, linguistic labels become inextricably linked to motor programmes through highly frequent co-occurrence in the input. These motor programmes are both the infant's own motor programmes, through their own interaction with objects, as well as the observed motor programmes in carers (which activate their own motor systems through mirror mechanisms). However, their account of the way in which the action system is involved in ‘generating’ meaning and language comprehension is more complex. Upon hearing a linguistic label for an object, the brain activates motor programmes associated with actions that have been associated with that object (through temporal co-occurrence). This activation generates predictions about effects (in the sense of sensori-motor consequences) of actual actions. Meaning is in effect generated from these predictions—i.e. from the expected outcomes of action.

Here, again, iconicity can provide an additional, mechanism for the grounding of language in sensori-motor systems. Under an embodied view of language, linguistic/communicative forms have meaning by virtue of being linked with real-world referents. Meaning is derived from mental simulations/representations of perceptual and motoric experience with real-world referents. Thus, iconic mappings, by their very nature of depicting perception-based and action-based properties of referents, imply the engagement of sensori-motor systems in processing the meaning of a linguistic signal. In grounding language in sensori-motor systems—through iconicity, as well as through mechanisms like Hebbian learning—it may well be that links between words and the world are made first for first-hand perceptual and motoric experience, and that structural alignment processes help to generalize to other, non-first-hand experiences once mental representations based on sensori-motor properties have been built up (see also [26]).

An embodied view of language stands in contrast to traditional views of language as a system of abstract symbol manipulation, which is separate from other aspects of perception, action and cognition. Iconicity makes links between linguistic/communicative forms and perception and action immediately clear. As such, it may be the case that embodied views of language would have gained popularity much earlier if the study of language had started with sign languages, where the multichannel and iconic nature of language is obvious, rather than with spoken languages. The relationship between iconicity and embodiment may thus be a demonstration *par excellence* of the overarching theme of this special issue—asking how our theoretical and methodological approaches to language should be informed by taking the multichannel and iconic nature of language as our starting point.

More generally, assuming that displacement and conceptual reference—as the most crucial adaptations of language as a system of communication—are achieved with the help of iconic signals, evoking the presence of a referent even in its absence, we provide a theoretically motivated reason for why sensori-motor systems would be involved in language. This is an important point, as any account of the phylogenesis and ontogenesis of language must also account for how the sensori-motor neural systems come to be engaged in language use. If this is the case, iconicity should have facilitatory effects in language processing as it would render the link between form and meaning stronger.

### (a) Iconicity effects in language processing

There are now a number of studies showing effects of iconicity in language processing (see Perniss *et al*. [13] for a more extensive review). In signed languages, Thompson *et al*. [109] found that processing of signs in signers of American Sign Language (ASL) is facilitated when the iconic link between a sign and its referent is highlighted. Signers performing a picture–sign matching task were faster to indicate that a sign referred to a previously viewed picture when the property of the referent iconically represented in the sign (e.g. tracing the cat's whiskers in the ASL sign for cat) was also highlighted in the picture (e.g. a picture of a cat's face with the whiskers prominent versus a picture of a whole cat). In another study, signers of BSL were slower in judging the phonological properties of signs (i.e. curved versus straight fingers) when signs were iconic compared to when they were non-iconic [23]. This finding is notable in that it suggests that the tight coupling of form and meaning in iconic signs leads to automatic activation of meaning, even when meaning is not necessary to performing the task (and it actually interferes with the task).

For spoken language, where iconicity is less abundant in the lexicon, much of the evidence for effects of iconicity on language processing comes from studies of vocabulary learning, where iconic mappings can be built into novel words. For example, Kovic *et al*. [110] found that adults who were asked to learn sound-symbolically congruent versus incongruent form–meaning associations, in a task learning labels for alien animal-like creatures, were faster to accept and slower to reject congruent form–meaning associations. Nygaard *et al*. [111] found that English speakers were better able to learn Japanese sound-symbolic words when they had been taught the correct English translation of the word compared to when they had been taught a semantically unrelated wrong translation of the word, suggesting that iconic, sound-symbolic mappings may reflect a more general cross-linguistic phenomenon. Evidence for a processing advantage of regular form–meaning mappings in spoken English comes from the study of phonaestemes (e.g. the association of /gl/ with a meaning of low light intensity, as in ‘glint’, ‘glitter’, ‘glow’, ‘glare’, or the association of /wr/ with a meaning of torqueing or distortion, as in ‘wreck’, ‘wrestle’, ‘writhe’, ‘wring’). While it is not clear whether these regular mappings embed actual *iconic* mappings, i.e. based on form–meaning resemblance, Bergen [112] demonstrated facilitated lexical access for phonaestemic form–meaning mappings, over and above the effects of phonological and semantic priming. In spoken languages, it is further the case that a mismatch between speech and iconic gestures (e.g. hearing the word ‘twist’ while watching a speaker making a gesture for ‘chopping’) slows down and induces more errors in comprehension, as would be expected if language comprehension implies integration of speech and gestures [8]. Finally, a number of neuroimaging experiments have shown engagement of sensori-motor cortices in the processing of language relating to the specific sensory and motor processes (e.g. [113,114] and see [107] for a review).

## 5. Possible criticisms

### (a) Language versus communication

A first possible criticism is that in expanding our view to language as a multichannel phenomenon and a system of face-to-face communication, we are no longer dealing with language *per se*, rather we end up concerning ourselves with those aspects of communication as human behaviour that are not central to language. It is certainly the case that we take a broad perspective on language, considering it as a system of human communication and interaction in contrast to the more familiar narrow perspective in which language is taken to be a linguistic system expressed in the rule-governed concatenation of morphological/lexical units (as is evident in speech or text).

Our broad perspective is motivated by the observation that language, as it is learnt, produced and understood, occurs primarily in face-to-face communicative contexts. As such, language includes information expressed in other channels and consists of more than a purely linguistic signal. The intrinsic difficulty in separating language from face-to-face communication becomes especially clear when we consider languages that can only be transmitted in a face-to-face situation, such as sign languages, but is just as relevant for spoken languages. In general, we would argue that current theories of language have been encumbered by too narrow a focus on the object of study, attempting to explain the emergence of an ultimately vocal and arbitrary system. However, to understand language in its multifaceted use as a system for meaning representation in communicative interaction, viable theories of language must take into account the availability and use of multiple channels (vocal and visual) and formats (iconic and arbitrary) of expression [6,115,116].

Thus, we would reject the notion that our approach focuses on aspects of communication that are not central to language because they cannot be readily formalized in terms of linguistic structure. Rather, our approach represents a more comprehensive approach to understanding language that takes into account all channels of communicative expression and the interactive nature of such expression (see also [117]).

Such a broad perspective crucially affords the possibility to develop novel hypotheses concerning the design features of language (from phylogenetic and ontogenetic perspectives) and to derive predictions for future studies. As we have spelled out in the sections above, our theoretical framework allows us to provide novel answers to long-standing questions about how communicative signals were able to refer to non-present entities (displacement) and how children solve the problem of referential ambiguity in learning their first language.

### (b) Iconicity remains negligible in language: arbitrariness is the ‘stuff’ of language

We have argued in this paper that iconicity is a critical feature of language, representing an adaptation to the fundamental constraint of language to link linguistic form to human experience. As such, iconicity has important implications for the three main areas of language study—evolution, learning and processing. In language evolution, iconicity achieves displacement—arguably the *design feature* of language that should be accorded primary status in jump-starting the communicative system that we now know as human language—and thus the ability for conceptual reference. In language learning, iconicity critically supports the referential mapping process by highlighting similarity between linguistic form and referent, and enables language learning episodes when referents talked about are not present. In language processing, iconicity achieves the engagement and grounding of our linguistic representations in our sensori-motor neural systems, what has come to be referred to as the embodiment of language. Thus, under our hypothesis, iconicity is a fundamental and crucial property of language that provides a means for achieving the fundamental referential function of language in each of these main domains. This view does not deny a critical role for arbitrariness. As argued in Perniss *et al*. [13], arbitrariness would also represent a central adaptation to a different constraint of language: the need for the linguistic signal to be efficient and discriminable [15,16,118].

This presents a possible criticism: it may be that iconicity plays an initial role in language evolution, providing the initial impetus for referential communication, but that it is dispensable to language as it exists in adults today. Here, iconicity would represent a mere remnant of a previous stage of language, a living fossil of proto-language [119], with arbitrariness representing the real stuff of language. For example, Dediu & Levinson [43] (p. 8) write: ‘the peculiarity of linguistic symbols is that they denote by abstract convention, while a cave painting of a horse denotes by iconic similarity, a principle that plays a very minor role in language’. The critic would thus hold that: in language evolution, iconicity might have helped in the development of displacement, but once this was initiated, the human ability to abstract from sensori-motor experience (hence to master arbitrary systems) took over and led the way to the development of our sophisticated linguistic system. Of course, this must also be the case to some extent. As adult language users, our mastery of sophisticated and highly abstract linguistic systems is notable, and as children (especially learning Indo-European languages), we learn substantial vocabularies that conform to the standard tenet of arbitrariness.

There are two critical points to make, however. First, if iconicity plays a pivotal role in establishing displacement in evolution, this fact already makes iconicity more than just a marginal phenomenon. Second, and more crucially, we would not expect effects of iconicity in language processing and acquisition if the role of iconicity were limited to jump-starting referentiality in evolution; however, we do find such effects of iconicity. As we have discussed, there is a growing body of evidence showing effects of iconicity in processing and acquisition.

Finally, there is still a different way in which iconicity may be argued not to reflect general properties of language. There is clearly a disproportionate amount of iconicity in sign languages in comparison to spoken languages. This may be taken by some to represent a modality difference between signed and spoken languages. Sign languages are still considered by many to not represent the ‘real stuff’ of language, but rather to demonstrate the fundamental flexibility and plasticity of the human cognitive system, reflecting the capacity for development of language in an alternate modality when acoustic sensory input is lacking. Under this view, iconicity (i.e. as a modality effect) may simply reflect adaptation to sensory deprivation. The burden, then, is for any defendant of such a position to explain how and why iconicity effects would be found in spoken languages at all. Moreover, they would further need to explain why a theory that assumes two independent explanations for iconicity effects in signed versus spoken language should be favoured over a more parsimonious theory that can account for all of these phenomena within a single framework.

### (c) But this is all to do with the lexicon, what about grammar?

Throughout this paper, we have discussed vocabulary, and whereas it is certainly the case that words are part of language, it is also the case that grammar is more often taken to represent the core of language. In particular, the property of recursivity in grammar has been taken to be the specific feature of hierarchical structure that marks human language out from other animal communication systems [120–122]. Our discussion of iconicity has pertained primarily to the lexicon, and not to grammar and the linguistic structure of language, i.e. the morphosyntactic organization of units of language. Though we have stressed throughout the need to define language as more than simply linguistic structure, our notion of language obviously also includes linguistic structure and grammar.

In terms of language development—in both phylogenesis and ontogenesis—grammatical/morphosyntactic structure would evolve later and more gradually than word forms and represents a higher level of complexity and abstraction [123]. This may give rise to the idea of a divide, or tension, between iconicity and grammar, as expressed recently e.g. by Meir *et al*. [124] (p. 310): ‘Iconicity is often depicted as a more basic representation device, while grammar supports the arbitrariness that comes with higher levels of symbolic processing’. However, iconicity has long played an important role in explanations of morphosyntax and grammar [118,123,125–127]. Thus, for spoken languages, the role of iconicity in the evolution of grammatical structure may be said to have a stronger, more established tradition compared to discussion of iconicity in the lexicon, with a large body of literature to support the general idea that the structure of language reflects the structure of experience. For example, the principle of ‘iconicity of sequence’ (or ‘sequential order’) holds that the sequence of forms conforms to the sequence of experience, as in the famous collocation *veni, vidi, vici*. The principle of ‘iconicity of contiguity’ (or ‘linguistic proximity’) assumes that forms that belong together conceptually and semantically will occur closer together morphosyntactically than forms that are conceptually and semantically unrelated (cf. Bybee's [128] analysis of the proximity relation between verb stem and inflectional categories according to conceptual relevance). For sign languages, the opposite may be true: iconicity in the lexicon has always been acknowledged, whereas descriptions of grammatical aspects of sign language structure included iconicity much later by comparison. The role of iconicity in structuring domains that rely on the use of space (e.g. pronouns, verbs) has been particularly acknowledged ([116,129,130]; see also Perniss [131] for a review). In most current approaches, structure in these domains is framed in terms of exhibiting a confluence of linguistic and ‘gestural’ (i.e. imagistic, iconic) elements—an effect of the visual modality's inherently iconic and spatial nature. However, recently, the role of iconicity in sign language structure has also been discussed in terms of what might be considered grammar *per se*, as part of the evolution of grammatical structure [124,132].

## 6. Conclusion

This paper has spelled out a theoretical view, in which iconicity plays a fundamental role in language development and language processing. The starting point for this proposal is the recognition that in order to further our understanding of language evolution, learning and processing and to move beyond our current state-of-the-art in language sciences, we must focus our attention on how language unfolds in face-to-face communication. Once we take such a perspective, iconicity appears as a widespread phenomenon in language. Iconicity, we argue, provides a key to how humans share sensory, motor and affective experiences with each other via communication.

Specifically, we argue that iconicity is at the root of three fundamental features of human language: the capacity for *displacement* during human evolution, the capacity to establish *referentiality* during language acquisition and the *embodiment* of adult language processing. Thus, we present a parsimonious and unified view on how linking linguistic form to human experience is achieved in evolution, development and processing.

There are many predictions to be tested from this theory. For example, a straightforward prediction concerning neural activation in language comprehension is that activation of areas associated with motor processing should be greater for signs exhibiting action-based iconicity or for speech accompanied by action-based co-speech gestures. Other predictions concern alignment of the developmental time course of perceptuo-motor skills in infants and toddlers with a corresponding time course of accessibility to different types of iconic mappings.

A major challenge for future research is to move beyond the holistic notion of iconicity that has guided research so far to a multidimensional notion that takes into account the type of iconic links (e.g. action-based versus perception-based) and the level of abstraction from actual experience, as we have discussed above. Moreover, any new conceptualization of iconicity will need to be viable across language modalities (signed and spoken) and across communication channels (in sign languages: hand, mouth and body; in spoken languages: speech, gestures and prosody).
